# Dimethyl 4,5-di­chloro­phthalate

**DOI:** 10.1107/S2414314621010439

**Published:** 2021-10-13

**Authors:** Daniel D. Hickstein, Eric W. Reinheimer, Adam R. Johnson, Daniel J. O’Leary

**Affiliations:** a Department of Chemistry, Pomona College, 645 N. College Ave., Claremont, CA 91711, USA; b Department of Chemistry and Biochemistry, W.M. Keck Foundation Center for Molecular Structure, California State University San Marcos, 333. S. Twin Oaks Valley Road, San Marcos, CA 92096, USA; c Department of Chemistry, Harvey Mudd College, 301 Platt Blvd., Claremont, CA 91711, USA; Howard University, USA

**Keywords:** crystal structure, carbon­yl, ester, metathesis, catalyst

## Abstract

While endeavoring to synthesize new chlorinated ligands for ruthenium-based metathesis catalysts, the title compound dimethyl 4,5-di­chloro­phthalate, C_10_H_8_Cl_2_O_4_, was prepared from commercially available 4,5-di­chloro­phthalic acid in ∼77% yield. The title mol­ecule, which also finds utility as a precursor mol­ecule for the synthesis of drugs used in the treatment of Alzheimer’s disease, shows one carbonyl-containing methyl ester moiety lying nearly co-planar with the chlorine-derivatized aromatic ring while the second methyl ester shows a significant deviation of 101.05 (12)° from the least-squares plane of the aromatic ring. Within the crystal, structural integrity is maintained by the concerted effects of electrostatic inter­actions involving the electron-deficient carbonyl carbon atom and the electron-rich aromatic ring along the *a*-axis direction and C—H**⋯**O hydrogen bonds between neighboring mol­ecules parallel to *b*.

## Abstract 2

The solid-state structure of dimethyl 4,5-di­chloro­phthalate is presented. One of the carbonyl-containing ester groups is nearly co-planar with the aromatic ring while the second deviates considerably from the least-squares plane of its chlorine-derivatized aromatic ring. Solid-state integrity is maintained by both electrostatic inter­actions and C—H**⋯**O hydrogen bonds.

## Graphical abstract

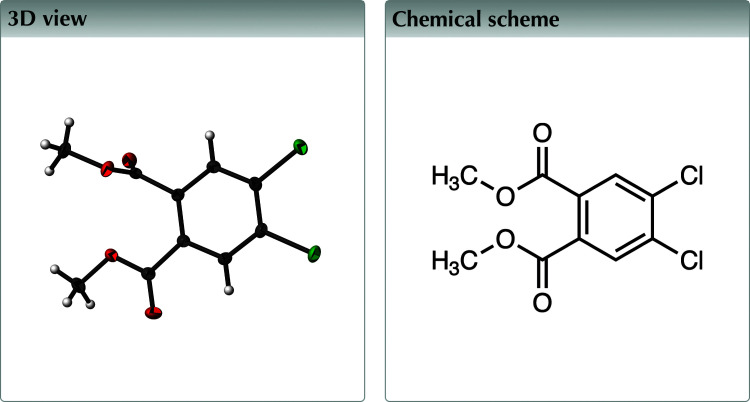

## Structure description

While endeavoring to synthesize new chlorinated ligands for ruthenium-based metathesis catalysts (Anderson *et al.*, 2006 ▸), the title compound, **1**, was prepared from commercially available 4,5-di­chloro­phthalic acid in ∼77% yield. The title mol­ecule also finds utility as a precursor mol­ecule for the synthesis of drugs used in the treatment of Alzheimer’s disease (Hennessy & Buchwald, 2005 ▸).

**Figure 1:**
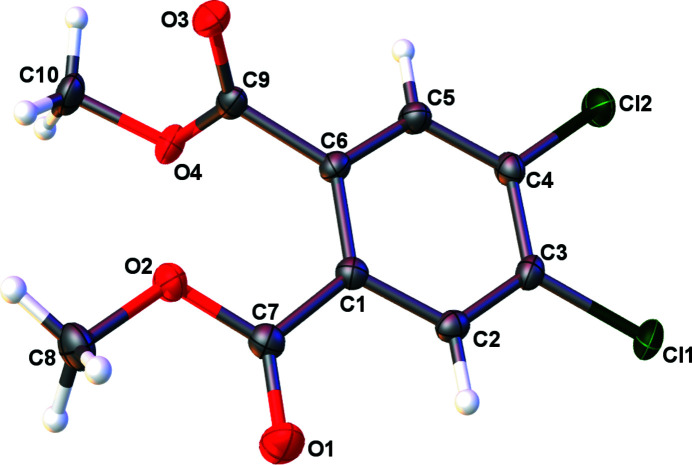
Anisotropic displacement ellipsoid plot of **1** with ellipsoids set to the 50% probability level.

Compound **1** crystallizes in the centrosymmetric triclinic space group *P* 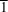 with a full mol­ecule of the title compound as the contents of asymmetric unit (Fig. 1 ▸, Table 1 ▸). Within the structure of **1**, one of the carbonyl-containing ester groups is nearly co-planar with the aromatic ring demonstrating a deviation of 3.41 (12)° from the least-squares plane of the chlorine-derivatized aromatic ring. The second ester group reveals a much larger deviation from planarity as the dihedral angle involving the second carbonyl group is 101.05 (12)°.

**Figure 2:**
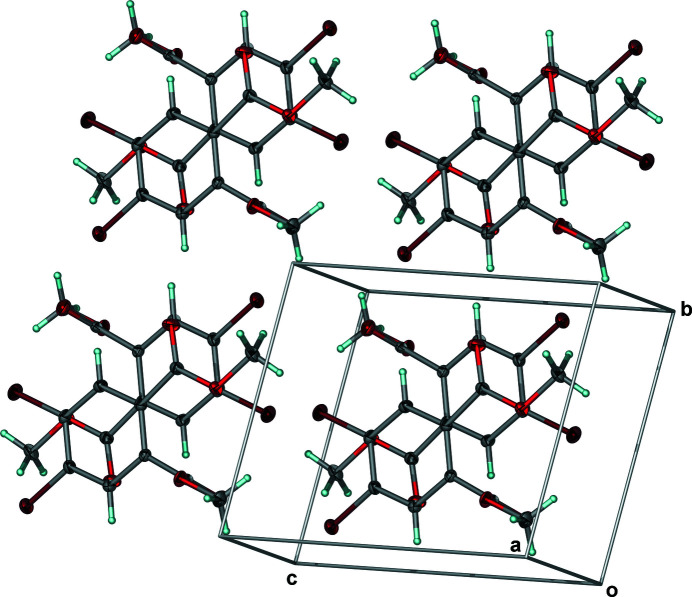
Solid-state expansion of **1** showing the superposition of the electron-rich aromatic ring centroid and the electron-deficient carbonyl carbon atom. Anisotropic displacement ellipsoids have been set to the 50% probability level.

**Figure 3:**
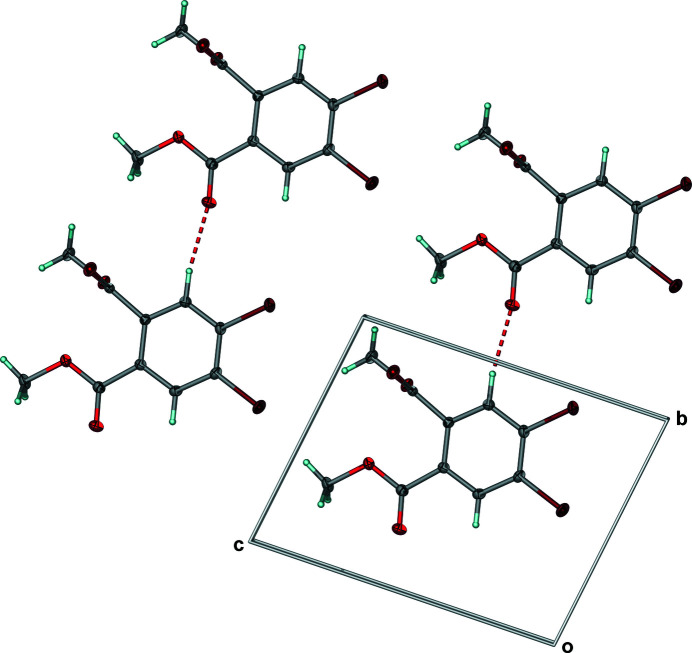
Projection of **1** within the *bc*-plane showing the C—H⋯O hydrogen bonding between neighboring mol­ecules along *b* to form one-dimensional arrays. Anisotropic displacement ellipsoids have been set to the 50% probability level. Dashed lines represent hydrogen bonds.

**Figure 4:**
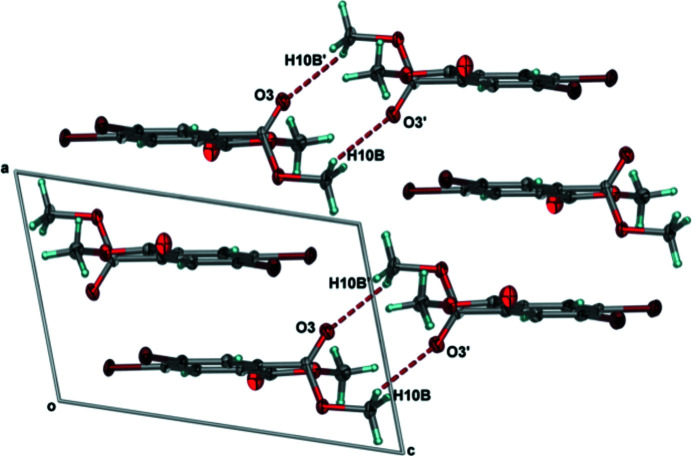
Projection of **1** within the *ac*-plane showing the formation of the 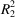 (10) centrosymmetric dimer facilitated by weak C—H⋯O inter­actions between layers. Anisotropic displacement ellipsoids have been set to the 50% probability level. Dashed lines represent the C—H⋯O inter­actions.

Looking down the *a*-axis, and involving a second mol­ecule of **1** related by inversion, the centroid of the electron-rich, chlorine-derivatized aromatic ring of the first mol­ecule lies above the electron-deficient carbonyl carbon atom of the second at a distance of 3.4600 (12) Å, suggesting the presence of electrostatic inter­actions (Fig. 2 ▸). In addition to the electrostatic inter­actions, when looking into the *bc*-plane, between H5 on the aromatic ring and O1 from the carbonyl that is nearly co-planar with the aromatic ring, a C—H⋯O [*d*(C5⋯O1) = 3.23 Å; *Θ*(C5—H5—O1) = 159°] hydrogen bond was observed (Fig. 3 ▸, Table 2 ▸). A one-dimensional array of symmetry-equivalent mol­ecules of **1** linked by C—H⋯O hydrogen bonds results along the *b*-axis direction when looking into the *bc*-plane (Fig. 3 ▸). While there are no additional inter­actions between neighboring, co-planar one-dimensional arrays parallel to one another along *c*, weak C—H⋯O [*d*(C10⋯O3) = 3.54 Å; *Θ*(C10—H10*B*—O3) = 147°] inter­actions with a neighboring layer having the symmetry code (1 − *x*, −*y*, −*z*) yielded a centrosymmetric dimer (Fig. 4 ▸, Table 2 ▸) having the 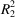 (10) graph-set notation (Bernstein *et al.*, 1995 ▸).

## Synthesis and crystallization

Compound **1** was synthesized by adding 4,5-di­chloro­phthalic acid (23.68 mmol, 5.566 g) to 70 ml of CH_3_OH in a 200 ml flask. While stirring, 1.0 ml H_2_SO_4_ (98%) was added dropwise and the mixture was allowed to reflux at 70°C overnight. The product was extracted with ethyl acetate, and washed with water, concentrated NaHCO_3_, 10% NaHCO_3_, and then a saturated solution of NaCl. After filtering through Na_2_SO_4_ to remove trace moisture, the solvent was removed *in vacuo* to yield a clear oil, which later crystallized into small rods. Recrystallization from the mixed solvents of isopropyl alcohol and di­chloro­methane produced X-ray quality crystals of **1** up to 2 mm.

## Refinement

Crystal data, data collection and structure refinement details for **1** are summarized in Table 2 ▸. The choice of the space group *P* 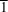 for **1** was unambiguously verified by *PLATON* (Spek, 2003 ▸; Spek, 2020 ▸).

## Supplementary Material

Crystal structure: contains datablock(s) I. DOI: 10.1107/S2414314621010439/bv4041sup1.cif

Structure factors: contains datablock(s) I. DOI: 10.1107/S2414314621010439/bv4041Isup2.hkl

Supporting information file. DOI: 10.1107/S2414314621010439/bv4041Isup3.cml

CCDC reference: 720360

Additional supporting information:  crystallographic information; 3D view; checkCIF report

## Figures and Tables

**Table 1:** Hydrogen-bond geometry (Å, °)

*D*—H⋯*A*	*D*—H	H⋯*A*	*D*⋯*A*	*D*—H⋯*A*
C5—H5⋯O1^i^	0.95	2.33	3.2327 (15)	159
C10—H10*B*⋯O3^ii^	0.98	2.68	3.5380 (16)	147

Symmetry codes: (i) 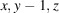 ; (ii) 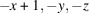 .

**Table 2:** Experimental details

Crystal data	
Chemical formula	C_10_H_8_Cl_2_O_4_
*M*_r_	263.06
Crystal system, space group	Triclinic, *P* 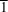
Temperature (K)	173
*a*, *b*, *c* (Å)	7.0204 (6), 7.7661 (6), 10.5392 (8)
α, β, γ (°)	97.733 (1), 109.293 (1), 90.217 (1)
*V* (Å^3^)	536.69 (7)
*Z*	2
Radiation type	Mo *K*α
μ (mm^−1^)	0.60
Crystal size (mm)	0.35 × 0.29 × 0.28

Data collection	
Diffractometer	Bruker *APEX* CCD area detector
Absorption correction	Multi-scan (*SADABS*; Krause *et al.*, 2015 ▸)
*T*_min_, *T*_max_	0.838, 0.927
No. of measured, independent and observed [*I* > 2σ(*I*)] reflections	5934, 2582, 2417
*R*_int_	0.031
(sin θ/λ)_max_ (Å^−1^)	0.668

Refinement	
*R*[*F* ^2^ > 2σ(*F* ^2^)], *wR*(*F* ^2^), *S*	0.026, 0.073, 1.04
No. of reflections	2582
No. of parameters	147
H-atom treatment	H-atom parameters constrained
Δρ_max_, Δρ_min_ (e Å^−3^)	0.45, −0.21

Computer programs: *APEX2* and *SAINT* (Bruker, 2016 ▸), *SHELXT2014/5* (Sheldrick, 2015*a* ▸), *SHELXL2014/7* (Sheldrick, 2015*b* ▸) and *OLEX2* (Dolomanov *et al.*, 2009 ▸).

## full crystallographic data

### Crystal data

**Table:**

C_10_H_8_Cl_2_O_4_	*Z* = 2
*M**_r_* = 263.06	*F*(000) = 268
Triclinic, *P*1	*D*_x_ = 1.628 Mg m^−^^3^
*a* = 7.0204 (6) Å	Mo *K*α radiation, λ = 0.71073 Å
*b* = 7.7661 (6) Å	Cell parameters from 548 reflections
*c* = 10.5392 (8) Å	θ = 2.4–27.7°
α = 97.733 (1)°	µ = 0.60 mm^−^^1^
β = 109.293 (1)°	*T* = 173 K
γ = 90.217 (1)°	Irregular, colorless
*V* = 536.69 (7) Å^3^	0.35 × 0.29 × 0.28 mm

### Data collection

**Table:**

Bruker APEX CCD area detector diffractometer	2582 independent reflections
Radiation source: Fine-focus sealed tube	2417 reflections with *I* > 2σ(*I*)
Graphite monochromator	*R*_int_ = 0.031
phi and ω scans	θ_max_ = 28.3°, θ_min_ = 2.1°
Absorption correction: multi-scan (SADABS; Krause *et al.*, 2015)	*h* = −9→9
*T*_min_ = 0.838, *T*_max_ = 0.927	*k* = −10→10
5934 measured reflections	*l* = −14→14

### Refinement

**Table:**

Refinement on *F*^2^	Primary atom site location: dual
Least-squares matrix: full	Hydrogen site location: inferred from neighbouring sites
*R*[*F*^2^ > 2σ(*F*^2^)] = 0.026	H-atom parameters constrained
*wR*(*F*^2^) = 0.073	*w* = 1/[σ^2^(*F*_o_^2^) + (0.0368*P*)^2^ + 0.1955*P*] where *P* = (*F*_o_^2^ + 2*F*_c_^2^)/3
*S* = 1.04	(Δ/σ)_max_ = 0.001
2582 reflections	Δρ_max_ = 0.45 e Å^−^^3^
147 parameters	Δρ_min_ = −0.21 e Å^−^^3^
0 restraints	

### Special details

**Table:**

Geometry. All esds (except the esd in the dihedral angle between two l.s. planes) are estimated using the full covariance matrix. The cell esds are taken into account individually in the estimation of esds in distances, angles and torsion angles; correlations between esds in cell parameters are only used when they are defined by crystal symmetry. An approximate (isotropic) treatment of cell esds is used for estimating esds involving l.s. planes.
Refinement. All non-hydrogen atoms were refined anisotropically. H atoms bound to C atoms were constrained to ride on the atoms onto which they are bonded, where C—H = 0.95 (aromatic) or 0.98 Å (methyl) with *U*_iso_(H) = 1.2*U*_eq_(C).

### Fractional atomic coordinates and isotropic or equivalent isotropic displacement parameters (Å^2^)

**Table:**

	*x*	*y*	*z*	*U*_iso_*/*U*_eq_	
Cl1	0.81953 (5)	0.48342 (4)	0.83259 (3)	0.02349 (9)	
Cl2	0.74478 (5)	0.08068 (4)	0.71750 (3)	0.02487 (9)	
O1	0.76916 (17)	0.76700 (12)	0.40055 (10)	0.0310 (2)	
O2	0.69277 (14)	0.55617 (11)	0.22210 (9)	0.02121 (18)	
O3	0.50933 (14)	0.17388 (12)	0.16137 (9)	0.02470 (19)	
O4	0.84744 (13)	0.20884 (11)	0.21752 (8)	0.02113 (18)	
C1	0.73795 (16)	0.47447 (14)	0.43804 (11)	0.0158 (2)	
C2	0.77075 (17)	0.53010 (15)	0.57530 (12)	0.0170 (2)	
H2	0.7904	0.6508	0.6094	0.020*	
C3	0.77483 (17)	0.41014 (15)	0.66231 (11)	0.0172 (2)	
C4	0.74445 (17)	0.23318 (15)	0.61230 (12)	0.0181 (2)	
C5	0.71216 (18)	0.17662 (15)	0.47593 (12)	0.0185 (2)	
H5	0.6921	0.0558	0.4423	0.022*	
C6	0.70903 (17)	0.29623 (14)	0.38820 (11)	0.0159 (2)	
C7	0.73570 (17)	0.61539 (15)	0.35369 (12)	0.0179 (2)	
C8	0.6829 (2)	0.68909 (17)	0.13612 (13)	0.0247 (3)	
H8A	0.6390	0.6352	0.0408	0.037*	
H8B	0.8168	0.7473	0.1603	0.037*	
H8C	0.5863	0.7746	0.1491	0.037*	
C9	0.67274 (18)	0.22206 (14)	0.24202 (12)	0.0175 (2)	
C10	0.8297 (2)	0.14662 (17)	0.07783 (12)	0.0249 (3)	
H10A	0.7640	0.2330	0.0200	0.037*	
H10B	0.7485	0.0365	0.0474	0.037*	
H10C	0.9646	0.1283	0.0717	0.037*	

### Atomic displacement parameters (Å^2^)

**Table:**

	*U*^11^	*U*^22^	*U*^33^	*U*^12^	*U*^13^	*U*^23^
Cl1	0.02835 (16)	0.02741 (16)	0.01490 (15)	−0.00009 (11)	0.00920 (12)	−0.00131 (11)
Cl2	0.03392 (18)	0.02289 (16)	0.01804 (15)	−0.00098 (12)	0.00730 (12)	0.00740 (11)
O1	0.0505 (6)	0.0152 (4)	0.0266 (5)	0.0004 (4)	0.0124 (4)	0.0022 (3)
O2	0.0281 (4)	0.0181 (4)	0.0175 (4)	−0.0002 (3)	0.0066 (3)	0.0052 (3)
O3	0.0253 (5)	0.0269 (4)	0.0174 (4)	−0.0058 (3)	0.0021 (3)	0.0010 (3)
O4	0.0235 (4)	0.0248 (4)	0.0136 (4)	0.0029 (3)	0.0056 (3)	−0.0005 (3)
C1	0.0142 (5)	0.0158 (5)	0.0169 (5)	0.0010 (4)	0.0045 (4)	0.0026 (4)
C2	0.0152 (5)	0.0161 (5)	0.0188 (5)	0.0008 (4)	0.0056 (4)	−0.0004 (4)
C3	0.0156 (5)	0.0217 (5)	0.0139 (5)	0.0011 (4)	0.0052 (4)	0.0000 (4)
C4	0.0183 (5)	0.0196 (5)	0.0164 (5)	0.0003 (4)	0.0049 (4)	0.0050 (4)
C5	0.0216 (5)	0.0155 (5)	0.0171 (5)	−0.0003 (4)	0.0048 (4)	0.0019 (4)
C6	0.0155 (5)	0.0165 (5)	0.0143 (5)	0.0002 (4)	0.0034 (4)	0.0011 (4)
C7	0.0168 (5)	0.0167 (5)	0.0204 (5)	0.0019 (4)	0.0059 (4)	0.0036 (4)
C8	0.0292 (6)	0.0237 (6)	0.0243 (6)	0.0038 (5)	0.0098 (5)	0.0115 (5)
C9	0.0242 (6)	0.0125 (5)	0.0150 (5)	0.0009 (4)	0.0049 (4)	0.0030 (4)
C10	0.0342 (7)	0.0261 (6)	0.0147 (5)	0.0029 (5)	0.0097 (5)	−0.0001 (4)

### Geometric parameters (Å, º)

**Table:**

Cl1—C3	1.7305 (12)	C2—C3	1.3865 (16)
Cl2—C4	1.7272 (12)	C3—C4	1.3931 (16)
O1—C7	1.2042 (15)	C4—C5	1.3871 (16)
O2—C7	1.3330 (15)	C5—H5	0.9500
O2—C8	1.4500 (14)	C5—C6	1.3915 (15)
O3—C9	1.2022 (15)	C6—C9	1.5069 (16)
O4—C9	1.3359 (15)	C8—H8A	0.9800
O4—C10	1.4503 (14)	C8—H8B	0.9800
C1—C2	1.3940 (16)	C8—H8C	0.9800
C1—C6	1.4018 (15)	C10—H10A	0.9800
C1—C7	1.4969 (15)	C10—H10B	0.9800
C2—H2	0.9500	C10—H10C	0.9800
			
C7—O2—C8	115.05 (9)	C5—C6—C9	116.26 (10)
C9—O4—C10	115.31 (10)	O1—C7—O2	123.60 (11)
C2—C1—C6	119.63 (10)	O1—C7—C1	123.11 (11)
C2—C1—C7	115.67 (10)	O2—C7—C1	113.29 (9)
C6—C1—C7	124.70 (10)	O2—C8—H8A	109.5
C1—C2—H2	119.8	O2—C8—H8B	109.5
C3—C2—C1	120.33 (10)	O2—C8—H8C	109.5
C3—C2—H2	119.8	H8A—C8—H8B	109.5
C2—C3—Cl1	119.14 (9)	H8A—C8—H8C	109.5
C2—C3—C4	119.92 (10)	H8B—C8—H8C	109.5
C4—C3—Cl1	120.94 (9)	O3—C9—O4	125.21 (11)
C3—C4—Cl2	121.06 (9)	O3—C9—C6	124.09 (11)
C5—C4—Cl2	118.80 (9)	O4—C9—C6	110.60 (10)
C5—C4—C3	120.14 (10)	O4—C10—H10A	109.5
C4—C5—H5	119.9	O4—C10—H10B	109.5
C4—C5—C6	120.23 (10)	O4—C10—H10C	109.5
C6—C5—H5	119.9	H10A—C10—H10B	109.5
C1—C6—C9	124.00 (10)	H10A—C10—H10C	109.5
C5—C6—C1	119.74 (10)	H10B—C10—H10C	109.5
			
Cl1—C3—C4—Cl2	1.42 (14)	C4—C5—C6—C1	−0.23 (17)
Cl1—C3—C4—C5	−178.93 (9)	C4—C5—C6—C9	−179.96 (10)
Cl2—C4—C5—C6	179.38 (9)	C5—C6—C9—O3	78.67 (15)
C1—C2—C3—Cl1	179.08 (9)	C5—C6—C9—O4	−97.92 (12)
C1—C2—C3—C4	−0.50 (17)	C6—C1—C2—C3	0.00 (17)
C1—C6—C9—O3	−101.05 (14)	C6—C1—C7—O1	−176.90 (12)
C1—C6—C9—O4	82.36 (13)	C6—C1—C7—O2	3.20 (16)
C2—C1—C6—C5	0.37 (17)	C7—C1—C2—C3	179.72 (10)
C2—C1—C6—C9	−179.92 (10)	C7—C1—C6—C5	−179.33 (10)
C2—C1—C7—O1	3.39 (17)	C7—C1—C6—C9	0.38 (18)
C2—C1—C7—O2	−176.51 (10)	C8—O2—C7—O1	−1.71 (17)
C2—C3—C4—Cl2	−179.00 (8)	C8—O2—C7—C1	178.18 (9)
C2—C3—C4—C5	0.65 (17)	C10—O4—C9—O3	6.33 (17)
C3—C4—C5—C6	−0.28 (18)	C10—O4—C9—C6	−177.12 (9)

### Hydrogen-bond geometry (Å, º)

**Table:**

*D*—H···*A*	*D*—H	H···*A*	*D*···*A*	*D*—H···*A*
C5—H5···O1^i^	0.95	2.33	3.2327 (15)	159
C10—H10*B*···O3^ii^	0.98	2.68	3.5380 (16)	147

Symmetry codes: (i) *x*, *y*−1, *z*; (ii) −*x*+1, −*y*, −*z*.
